# The association of negative mood with automatic and effortful facial expression mimicry

**DOI:** 10.3389/fpsyg.2023.1056535

**Published:** 2023-04-24

**Authors:** Tara L. Kraft-Feil, Rick E. Ingram, Claire Gorey, Jazlyn H. Luu, Marie P. Cross, Sarah D. Pressman

**Affiliations:** ^1^CHI St. Alexius Health, Bismarck, ND, United States; ^2^Department of Psychology, University of Kansas, Lawrence, KS, United States; ^3^Department of Psychology, University of South Florida, Tampa, FL, United States; ^4^Department of Psychological Science, University of California, Irvine, Irvine, CA, United States; ^5^Department of Biobehavioral Health, Pennsylvania State University, University Park, PA, United States

**Keywords:** facial expression, mimicry, negative mood, negative emotion, negative affect, electromyography, loneliness, muscle activation

## Abstract

The natural process of mimicking the facial expressions of others is well established, as are the deficits in this reflexive behavior for individuals with clinical disorders such as depression. This study examines the extent of this deficit in non-clinical individuals with high transient negative mood, and whether it extends to both automatic and effortful emotion expression behavior. One hundred and thirty-six participants were shown happy, sad, and neutral faces, while electromyography (EMG) recorded facial muscle responses. Automatic (reflexive) mimicry was assessed while participants simply viewed facially expressive photographs, while effortful mimicry was monitored when individuals were told to intentionally copy the expressions in the photographs. Results indicated that high levels of negative mood were primarily associated with deficits in effortful mimicry of happy expressions, although some similar evidence was found in automatic mimicry of happy faces. Surprisingly, there were also ties between negative moods and inaccuracies in effortful mimicry of sad expressions (but not automatic mimicry). Inaccurate automatic and effortful mimicry were also tied with lower self-reported social support and greater loneliness. These results indicate that even in healthy individuals, transient and minor changes in negative mood are tied to deficiencies in facial mimicry at both the automatic and effortful level.

## Introduction

Facial expression plays a crucial role in human communication and social interaction. The ability to automatically (unconsciously) mimic or imitate the facial expressions of others is a fundamental aspect of social behavior and is linked to various outcomes such as liking, dyadic rapport, emotional contagion, and the perception and interpretation of emotions (Vaughan and Lanzetta, 1981; Brothers, 1990; Hatfield et al., 1992; Cappella, 1993; Lundqvist and Dimberg, 1995). Unfortunately, this critical mimicking reflex is impaired in individuals with clinical disorders such as depression (Sloan et al., 2002; Zwick and Wolkenstein, 2017), alexithymia (Schiano Lomoriello et al., 2021), and other disorders (McIntosh et al., 2006; Varcin et al., 2010; Peter-Ruf et al., 2017; Passardi et al., 2019; Ziebell et al., 2021). There is even evidence that non-clinical samples with negative moods (Ingram and Hamilton, 1999) also have disrupted automatic mimicry (Sloan et al., 2002; Lautzenhiser, 2003), pointing to the possibility that even mild negative mood (NM) can disrupt mimicry behavior.

While less studied in this manner, research has also highlighted the importance of effortful (conscious) mimicry in social connection and well-being. For example, Tuck et al. (2016a) found that this type of intentional mimicry in social interactions has positive effects on outcomes such as liking, trust, cooperation, and attraction. Additionally, effortful mimicry has been found to improve the ability to understand the emotions of others (Lewis and Dunn, 2017). These findings point to the need for future research on the connections between NM and *both* types of mimicry, particularly in non-clinical samples. Understanding the connections may provide insight into the social deficits associated with NM and inform the development of interventions such as facial expression training programs (Segrin, 2000) that target these deficits for the general population.

As will be discussed below, effortful and automatic mimicry are separate phenomena that are thought to involve different neural and cognitive mechanisms (Chartrand and Bargh, 1999; van Baaren et al., 2004). Effortful mimicry refers to the conscious and deliberate imitation of the actions or expressions of others, whereas automatic mimicry refers to unconscious and spontaneous imitation. Research has shown that while both types of mimicry can lead to positive social outcomes (Chartrand and Bargh, 1999; van Baaren et al., 2004), automatic mimicry may be more closely related to the spontaneous and unconscious processes that underlie social cognition, while voluntary mimicry may be more closely related to the conscious and controlled processes that underlie social behavior. Therefore, examining both types of mimicry in one study allows for a more comprehensive understanding of the underlying mechanisms and provides a way to test the relative importance of these different forms of mimicry in the context of NM, and how they interact with each other.

### Types of mimicry

Automatic (also called unconscious or reflexive) mimicry occurs subconsciously and rapidly, typically within 1,000 ms after viewing a facial expression, whereas effortful mimicry (also called intentional or voluntary) involves a slower, conscious effort to replicate another’s facial expression (Bargh and Williams, 2006). While these two types of mimicry are both forms of imitation, they differ fundamentally from both a theoretical and functional perspective and should be regarded as separate phenomena. Automatic mimicry happens without awareness and is considered an innate aspect of human nature that plays a key role in social interactions (Schneider et al., 2013; Prochazkova and Kret, 2017). In fact, automatic mimicry of others’ facial expressions can be seen as early as a few days after birth, with infants mimicking happy and sad faces of their caregivers (Field et al., 1982). Theories of automatic facial mimicry propose that this behavior serves an adaptive function, allowing individuals to understand and respond to the emotions of others, as well as to convey their own emotions more effectively. For example, the “emotional contagion” theory suggests that automatic mimicry allows individuals to “catch” the emotions of others, leading to a shared emotional experience (Hatfield et al., 1992). The “social tuning” theory, on the other hand, proposes that automatic mimicry serves as a way to build rapport and establish social connections with others (Chartrand and Bargh, 1999). Recent findings have further highlighted the importance of automatic mimicry in social interactions. For example, Prochazkova and Kret (2017) found that individuals who displayed higher levels of automatic mimicry also reported higher levels of empathy and affiliation with others. Further, Stel and Vonk (2010) found that individuals who displayed higher levels of automatic mimicry also reported more positive social interactions such as greater trust, cooperation, and liking from others.

Effortful facial mimicry, or the deliberate act of copying another person’s facial expression (e.g., being explicitly told to mimic a photograph), is primarily distinguished from automatic mimicry as a conscious (versus unconscious) process. Laboratory studies have found evidence that this type of mimicry can be influenced by cognitive, social, and emotional factors (e.g., see review by Hatfield et al., 2014), and that this ability, like automatic mimicry, can be influenced by certain types of disorders (e.g., Parkinson’s disease; Kang et al., 2019). Moreover, Arnold and Winkielman (2021) also found that loneliness is associated with impaired spontaneous smile mimicry, highlighting the importance of investigating intentional mimicry as a social behavior in addition to unconscious mimicry processes. These studies, as well as others connecting the ability to mimic and express specific emotions on command to future mental and physical health (Tuck et al., 2016b, 2017), demonstrate the importance of understanding the mechanisms underlying intentional facial mimicry. While clearly both automatic and effortful mimicry have social value, effortful mimicry likely requires a certain amount of attentional availability and is thought to be used more strategically than unconscious mimicry (e.g., to persuade or deceive). Effortful mimicry could still serve social connection building purposes (Chartrand and Bargh, 1999), however, especially as it may allow individuals to better recognize the emotions in others (Niedenthal, 2007; Sato et al., 2013).

It is also important to make the distinction between emotional mimicry and behavioral mimicry. Emotional mimicry involves imitating the emotional expression of another and serves as a social signal that holds information about one’s appraisal of an event (Fischer and Hess, 2017). Furthermore, this form of mimicry often signals intentions and is considered a social regulator (Duffy and Chartrand, 2015; Hess and Fischer, 2022). For instance, emotional mimicry of happiness might hold an affiliative meaning, while mimicking anger or disgust might hold a more antagonistic meaning. On the other hand, behavioral mimicry involves the imitation of mannerisms, postures, gestures, and motor movements of another (Duffy and Chartrand, 2015). While this type of mimicry also serves social functions, it involves mimicking movement rather than emotional expressions. Thus, because this paper is focused on mimicry of facial expressions, we focus solely on emotional mimicry.

### Theories and research on the role of facial expression mimicry

Facial expressions are considered an important objective signal of underlying emotions. They have been recognized as instinctive and past research has demonstrated that there are universal facial expressions of various emotions such as happiness, sadness, anger, disgust, fear, contempt, and surprise (Darwin, 1965; Ekman, 1992). Modern research confirms that human facial expressions do serve as indicators of emotional experience, conveying emotion and eliciting emotions in others (Ruys and Stapel, 2008).

When considering the whys and effects of mimicry, there are many competing and complementary theories. For example, the “facial feedback hypothesis (FFH)” proposed by Tourangeau and Ellsworth (1979) suggests that physical change in the body (including facial musculature) could impact emotional experience, with facial muscle movements being an important part of constructing the emotional experience. Although the FFH has been the center of debate due to failed replications (Wagenmakers et al., 2016; Söderkvist et al., 2018), a recent large-scale meta-analysis and a multi-site international pre-registered study of the facial feedback phenomenon show that there is a replicable FFH effect, albeit a small one (Coles et al., 2019, 2022). Also relevant when considering the role of mimicry, and in particular how it may influence recognition, are the generate-and-test model and the reverse simulation model (Goldman and Sripada, 2005). The generate-and-test model highlights the existence of two processes: one with and one without mimicry. In the case of mimicry, the observer imitates the facial expressions of others in order to understand their emotions. If the copied expression matches the observed emotion, then the emotion will be classified accordingly. This theory suggests that mimicry plays a crucial role in understanding others’ emotions.

Connected to the generate-and-test model, research shows that mimicry of emotional expressions can improve facial affect sensitivity, or the ability to accurately interpret and respond to the emotions of others. For example, Schneider et al. (2013) found that individuals instructed to suppress their facial expressions showed impairment in facial affect sensitivity. In contrast, when participants were asked to produce a facial expression congruent with the observed one, greater accuracy was achieved. This suggests that mimicry of emotional expressions plays an important role in facilitating the ability to understand and respond to the emotions of others. Other studies have also supported these findings, such as a study by Strack et al. (1988) that found that participants who were able to mimic the expression of others were better able to identify the emotion being expressed. A more recent study investigating event-related potential markers of visual working memory within the context of facial mimicry determined that the quality of visual perception in memory is higher when individuals use facial mimicry (Sessa et al., 2018). In addition, the cortical circuits involved in the processing of emotional expressions may be different if individuals cannot use facial mimicry mechanisms, such as those who have congenital facial palsy (Sessa et al., 2022). Taken together, these findings highlight the importance of mimicry in social interactions and emotional understanding.

Interestingly, there is debate surrounding the specific roles and mechanisms of mimicry. While some theories propose that mimicry plays a large role in emotion recognition and contagion (Prochazkova and Kret, 2017; Winkielman et al., 2018), other theories focus on how mimicry fosters affiliation and builds rapport (Hess and Fischer, 2022) or plays a crucial role in visual emotion discrimination and perceptual processing of expressions (Wood et al., 2016). The role that mimicry plays in emotion recognition is unclear and findings are mixed. Studies with designs that block mimicry discover mediation, while studies without manipulations blocking mimicry find no relationship (Hess, 2021; Hess and Fischer, 2022). There are also mixed results from studies attempting to test the theory of mimicry mediating emotion contagion, where some find no relationship (Hess and Blairy, 2001; Lishner et al., 2008) and some find partial indirect effects (Lischetzke et al., 2020; Olszanowski et al., 2020), meaning other mechanisms besides mimicry may be responsible for emotion contagion during social interactions. Thus, while mimicry is undoubtedly influenced by social context (Prochazkova and Kret, 2017; Winkielman et al., 2018; Hess and Fischer, 2022), there is much debate surrounding the specific roles and mechanisms of mimicry.

### Measuring facial expressions via muscle activity in the face

**Figure 1:**
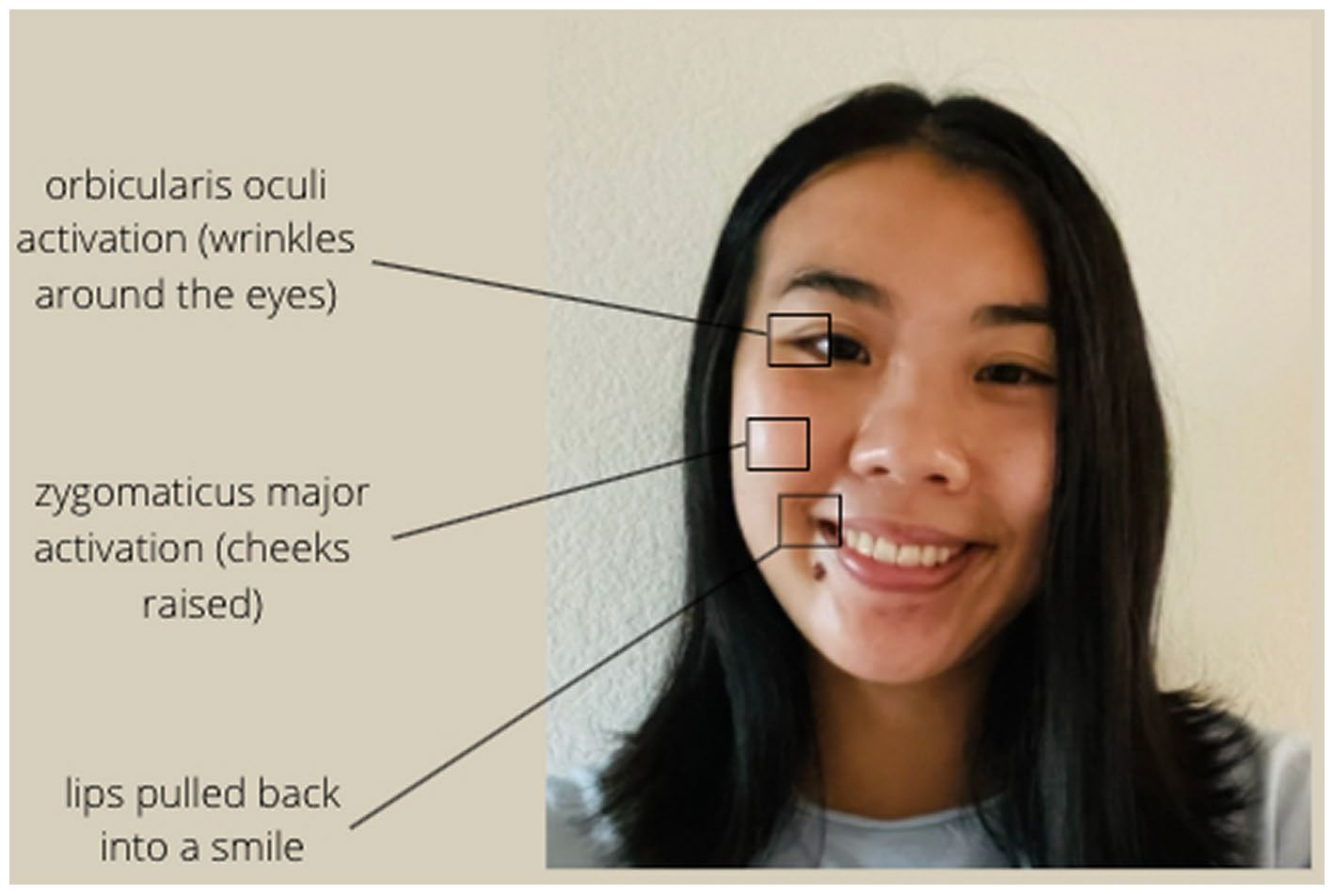
Muscles activated in Duchenne smiles.

**Figure 2:**
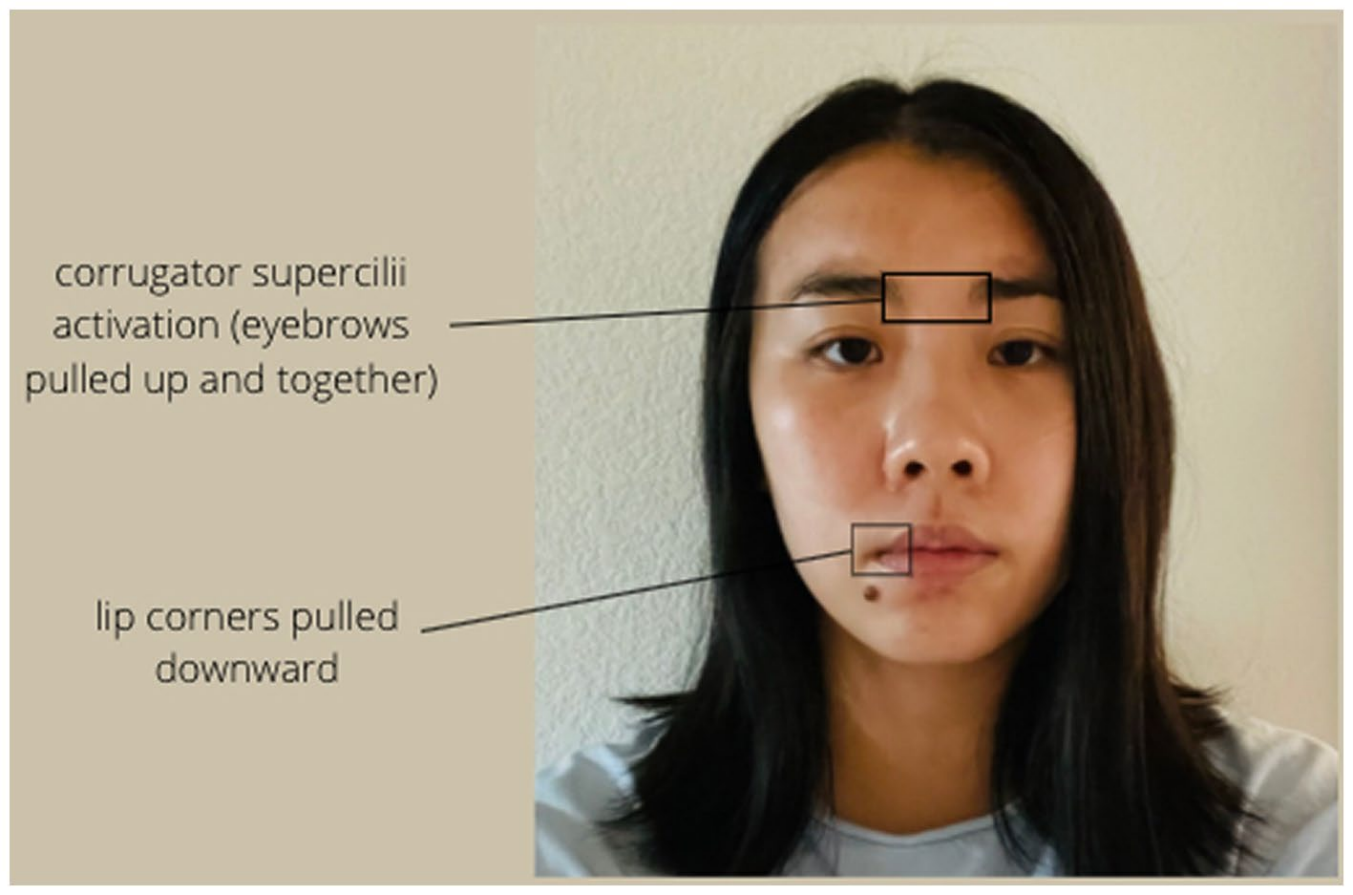
Muscles activated in frowns.

Facial expression is typically coded by examining differences in muscular activity in the face. Positive facial expressions include Duchenne smiles (Ekman and Friesen, 1982; Duchenne, 1990), which have been shown to be genuine signals of positive mood (Sheldon et al., 2021) and are typically recognized by activity in the zygomaticus major (Zygo) muscles in the cheeks, as well as the orbicularis oculi (Orbic) muscles around the eyes (see Figure 1). Sad facial expressions include frowning, which involve activation in the corrugator supercilii (Corr) muscles above the eyebrows (see Figure 2; Ekman and Friesen, 1978).

While there are many possible ways to code facial expressions, electromyography (EMG) is frequently utilized to detect difficult to observe fine and subtle muscle movements associated with facial expressions occurring in real time (see Tassinary and Cacioppo, 2000). EMG data are collected by placing surface electrodes on the skin above various muscle regions with wires attached to an amplifier in order to detect electrical activity moving across a particular muscle region. EMG has been shown to be a valid and reliable indicator of muscle activity associated with positive and negative facial expressions and of emotional experience in general (Winkielman and Cacioppo, 2001; Cacioppo et al., 2008). Furthermore, EMG has been widely used to identify minor (unobservable) muscle movement associated with automatic mimicry of others’ positive and negative facial expressions (Dimberg, 1982, 1990; Lanzetta and Englis, 1989).

### How depression and negative mood interact with mimicry and social functioning

As discussed above, mimicry of facial expressions plays a crucial role in effective social communication and feelings of social connectedness (Chartrand and Bargh, 1999; Arnold and Winkielman, 2021). However, individuals experiencing NM, such as depressed and dysphoric populations, have been found to have impaired mimicry reflexes (Sloan et al., 2002; Zwick and Wolkenstein, 2017). This can be explained by both cognitive and interpersonal theories, which suggest that negative patterns of thinking and difficulty with social functioning, common in depressed and dysphoric individuals, contribute to the impairment of processing and mimicking facial expressions (Lewinsohn, 1974; Cozolino, 2002; Bistricky et al., 2011).

Studies have found that dysphoric children and adults have difficulty processing and mimicking positive facial expressions, as compared to non-dysphoric individuals (Wexler et al., 1993; Sloan et al., 2002; Lautzenhiser, 2003). Research on cognitive processing of clear and ambiguous facial expressions has revealed differences in the way individuals process negative and positive faces, with negative faces being processed quickly and automatically, while positive faces are processed less quickly and with more effort (White, 1995). Individuals experiencing depression and dysphoria have been found to have a deficiency in processing positive expressions, which are processed effortfully, but not negative expressions, which are processed automatically (Hartlage et al., 1993; Yoon et al., 2016).

EMG studies have also found that depressed individuals exhibit less smiling in response to positive stimuli and more frowning in response to neutral stimuli (Lautzenhiser, 2003). Similarly, Sloan et al. (2002) found that while dysphoric individuals did not differ in automatic mimicry of sad faces, they did differ in automatic mimicry of happy faces, with non-dysphoric individuals showing expected cheek muscle activity while dysphoric individuals did not. Additionally, research by Wexler et al. (1993) found that while dysphoric individuals performed equally well on tasks of facial expression recognition, they did not show expected changes in facial muscle activity in response to sad or positive expressions. Furthermore, a study by Moody et al. (2007) suggests that rapid facial muscle mimicry can change based on an individual’s mood state, with participants showing less automatic mimicry in response to scary images. Overall, these findings highlight the impact of NM states (albeit typically studied in depressed/dysphoric individuals) on the ability to mimic and process facial expressions, and its potential impact on social functioning.

### Purpose of current study

This is an exploratory study examining whether NM in a healthy sample is associated with poor automatic and effortful mimicry of others’ facial expressions. Because past studies have not focused on both automatic and effortful mimicry with transient mood previously, we took a wide lens and explored many negative affect measurement types as they connected to three different facial expression types (happy, sad, and neutral) as well as three different facial muscles (orbicularis oculi, zygomaticus major, and corrugator supercilii). Based on past clinical depression research, we hypothesized that NM (broadly defined) would be particularly associated with decreased ability to both automatically and effortfully mimic positive facial expressions but did not expect strong associations with the ability to mimic neutral or negative facial expressions. We had no specific hypotheses on which types of NM would be most tied to mimicking outcomes. Next, given the observed ties between socially disconnected clinical samples and mimicry ability (e.g., in autistic samples; Yoshimura et al., 2015; Forbes et al., 2016), as well as indications in healthy samples of connections between facial expression skill and social connections (Seibt et al., 2015; Arnold and Winkielman, 2021), we hypothesized that greater accurate facial muscle activation change (from rest) during both automatic and effortful mimicry would be associated with higher self-reported social support and lower loneliness.

## Methods

### Participants

One hundred thirty-six college students were recruited through the online recruitment system of a large Midwestern university (see Table 1 for sample characteristics). The University of Kansas Institutional Review Board approved this study. Participants signed an informed consent prior to participation in a study called “Understanding Facial Expression.” They were made aware that they would be assessed by facial physiological monitoring, observing photos, and answering a series of self-report questions.

**Table 1:** Sample characteristics.

Variable		*n*	%	Range	*M (SD)*
Sex	Female	89	65.4		
	Male	45	33.1		
	No data	2	1.5		
Race					
	Caucasian	104	76.5		
	African American	8	5.9		
	Native American	2	1.5		
	Asian/Pacific Islander	8	5.9		
	Hispanic/Latino	7	5.1		
	Other	4	2.9		
	No data	3	2.2		
Age				18–30	19 (1.63)
Current psychological disorder					
	None	127	93.4		
	ADHD	1	0.7		
	Anxiety	3	2.2		
	Depression	2	1.5		
	Bipolar	1	0.7		
	Other	2	1.5		
Beck Depression Inventory scores				0–46	7.7 (8.0)
Pre-study baseline negative emotion				0.1–2.9	1.2 (0.7)

#### Inclusion criteria

Only fluent English speakers were eligible for participation. All participants with facial muscular disorders or injury were excluded to ensure that all participants were physically capable of mimicking facial expressions. All individuals reporting a psychological disorder were carefully examined to ensure that their responses did not inappropriately skew scores on dependent variables of interest.

### Measures

#### Demographic covariates

Basic demographic data, including age, sex, and race, were collected by self report prior to participation in the EMG component of the study.

#### Negative mood

NM was measured through the use of three scales/subscales. Two of these subscales were from a brief 24-item version of the Profile of Mood States (POMS) based on a factor analysis of original POMS items (McNair et al., 1971; Usala and Hertzog, 1989; Jenkins et al., 2021). The scale ranged from 0 to 4 for each item, with 0 indicating that the item was not at all accurate in describing how the participant felt, and 4 indicating that the item was extremely accurate in describing how the participant felt. Participants were asked about both emotion (i.e., what feelings were present “right now”) and mood (i.e., what feelings were present “during the past week”). There were five categories of subscales: Fatigue (including sluggish, tired, and sleepy), Depression (including unhappy, depressed, and sad), Fear (including fearful, frightened, and afraid), Anxiety (including on edge, nervous, and tense), and Hostility (including hostile, angry, and resentful). Due to the large literature on depression and mimicry discussed above, the current analyses focused only on Depression subscale scores (depressed emotion [DE] and depressed mood [DM]), in addition to the general negative emotion (NE) and general NM score (aggregate of all five subscales). Reliability (α ranging between 0.66–0.95) and validity of various short forms of the POMS have been established (McNair et al., 1971; Grove and Prapavessis, 1992).

The other scale through which NM was measured was the Beck Depression Inventory – Second Edition (BDI-II; Beck et al., 1996), which assesses dysphoric mood and symptoms of depression outlined in the Diagnostic and Statistical Manual of Mental Disorders Fourth Edition (DSM-IV-TR; 1994). Each item on the BDI-II assesses the existence and severity of a different symptom on a scale from 0 to 3, with 3 being the most severe. Scores range from 0 to 63, with scores between 13 and 19 indicative of dysphoric mood, and scores between 20 and 63 indicative of likely depression (Dozois et al., 1998). Reliability (α = 0.91) and validity of the BDI-II have been established (Dozois et al., 1998).

#### Social support

The Interpersonal Support Evaluation List-12 (ISEL-12; Cohen et al., 1985) is a 12-item self-report scale measuring perceived availability of social support. Individuals are asked to respond on a scale from 0 (Definitely False) to 3 (Definitely True) whether statements describing availability of varying types of social support (i.e., appraisal, belonging, self-esteem, and tangible) are true for their life experience. Sample items include, “If I were sick, I could easily find someone to help me with my daily chores” and “When I need suggestions on how to deal with a personal problem, I know someone I can turn to.” Reliability (α = 0.77) and validity of the ISEL-12 have been established (Cohen and Hoberman, 1983).

#### Loneliness

The UCLA Loneliness Scale-8 (Hays and DiMatteo, 1987) is an 8-item self-report questionnaire measuring feelings of loneliness or social isolation. Individuals indicate the degree to which they have experienced a series of statements on a scale from 1 (Never felt this way) to 4 (Always felt this way). Example items include “I lack companionship” and “There is no one I can turn to.” Internal reliability (α = 0.84) and validity have been well established (Hays and DiMatteo, 1987).

#### Experimental stimuli

Images of happy, sad, and neutral facial expressions were selected from the Karolinska Directed Emotional Faces (KDEF) system, a validated system of images containing 35 adult Caucasian females and 35 adult Caucasian males with various facial expressions (Lundqvist et al., 1998). Caucasian faces were chosen for this study because few standardized image sets with racially diverse images were available, and among those that were available, picture quality was substantially lower. Furthermore, most participants were Caucasian. Thus, the current study utilized five different male and five different female images portraying each facial expression from the KDEF image set. All individuals appearing in the images wore a gray t-shirt and were free of makeup. All images were selected from the 20 most validated KDEF images from each emotional expression category of interest and the 20 most validated individuals appeared in pictures across emotion categories (Goeleven et al., 2008). Furthermore, images were tested and selected by a team of 12 trained research assistants who unanimously agreed that the final set of images displayed the appropriate intended emotion.

#### Facial EMG data acquisition, reduction, and calculations

EMG data collection and processing followed psychophysiological standards (Tassinary and Cacioppo, 2000), with facial muscle movement measured continuously throughout the study and reported in volts (V) throughout the manuscript. Facial EMG signals were obtained from seven 4-mm electrodes placed on the left side of the face (see Fridlund and Cacioppo, 1986) using a MindWare BioNex EMG amplifier. One electrode was placed on the forehead as a ground electrode, and pairs of electrodes were placed approximately 1.25 cm apart on the muscle areas of interest: Corrugator Supercillii (Corr) muscle in the forehead, the Zygomaticus Major (Zygo) muscle in the cheek and the Orbicularis Oculi (Orbic) muscle around the eye. All sites for electrode placement were cleaned first with an alcohol swab and then with a gentle exfoliant in order to reduce impedance of electrode sites to less than 10 ohms. Data acquisition and recording of EMG was carried out using a MindWare BioLab 3.0 acquisition system. The signals were filtered with a low pass of 200 Hz and a high pass of 20 Hz and were sampled at 1000 Hz, with mean EMG levels calculated for each of the relevant epochs. Prior to final analyses, raw EMG data were screened and cleaned for electrical noise and movement artifacts (e.g., sneezing, coughing, yawning) and outliers greater than three standard deviations from the participant mean were removed. No more than 5% of observations were removed from any participant using these methods.

Data change score calculation procedures (relevant to baseline activity) are as follows: To assess whether NM was associated with the ability to automatically and effortfully mimic others’ facial expressions, change scores were calculated for each individual image by subtracting the mean facial muscle activation of the 4 s preceding the presentation of an image from the first 4 s the image was presented, since greatest affect-specific responses occur during the first 4 s of image presentation (see Wexler et al., 1993). Change scores for each image presentation were averaged together per block of facial expressions in order to obtain an average change in facial muscle activity for both automatic and effortful mimicry following sad, happy, and neutral faces separately. 4 s was chosen as the unit of averaging to accurately capture both effortful and automatic expression responses to the emotion stimuli. While past EMG work indicates that the *start* of mimicry can occur faster than 0.5 s post stimuli (Dimberg and Thunberg, 1998; Tarantili et al., 2005) and that healthy samples complete automatic mimicry of smiles in as little as 2 s (Krumhuber et al., 2014), classic research on smiling shows that natural smiles last for as long as 4 s (Ekman and Friesen, 1982) and that voluntary smiles are slower than what is observed in reflexive mimicry (Dimberg et al., 2002). Recent EMG work examining both effortful and automatic mimicry in the same study assessed out to 3.5 s post stimuli (Kang et al., 2019), and past emotion research has indicated that the greatest emotion specific responses occur in the first 4 s of image presentation (Wexler et al., 1993), indicating good convergent rationale for our selected time average.

### Procedure

Participants completed one 2-h session. Once consent and eligibility were verified, electrodes were placed as discussed above. Participants then sat in front of a computer monitor where they were asked to complete self-report measures. Once those were complete, participants were asked to sit quietly for a five-minute initial resting period. Facial muscle activity was assessed throughout the study. After the initial rest, participants were asked to view a series of images with individuals expressing a neutral, sad, or happy face. They were first instructed to simply watch the pictures in order to assess automatic muscle movement in reaction to the stimuli. Expressions were presented in 10-image blocks, with all images from the same emotional expression shown together (i.e., 10 happy, then 10 sad, then 10 neutral images; see McIntosh et al., 2006). The order of the images was randomized by block (i.e., some participants saw 10 happy, 10 sad, then 10 neutral, while others saw 10 neutral, 10 happy, then 10 sad). All possible combinations of block randomization (happy, sad, neutral; happy, neutral, sad; neutral, happy, sad; neutral, sad, happy; sad, happy, neutral; and sad, neutral, happy) were used to ensure randomization of all image sets across participants. Specifically, during all three 10-image blocks, each image was presented for 12 s, and another 12-s intertrial interval (ITI) appeared between images, with instructions to “watch the pictures as they appear on the screen” presented on the computer monitor in front of the participant during the ITI period. Between each 10-image set, participants were given questions that assessed emotions experienced, and a brief distractor task, which asked participants to memorize a 6-item grocery list and answer a brief question about whether a particular item was on the list. Rest periods lasted 5 min and facial muscle activity continued to be assessed during this time. This was completed to ensure that condition effects did not carry over from one condition block to the next.

**Figure 3:**
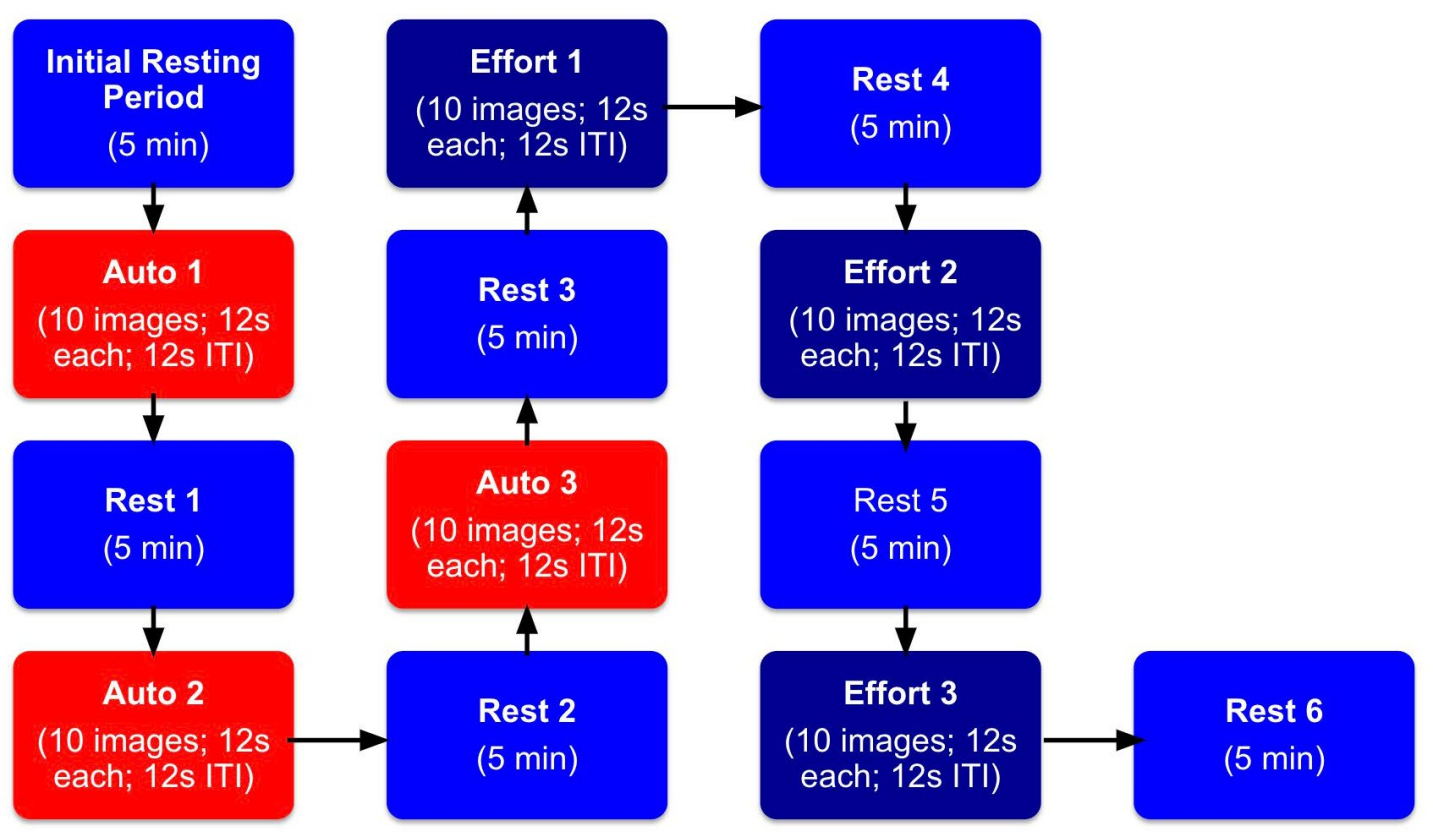
Facial mimicry experimental procedure. Blocks labeled “Initial Resting Period” indicate the initial resting period when participants were asked to sit quietly for 5 min. Blocks labeled “Rest” indicate when participants took a 5 min rest period in between the tasks. Blocks labeled “Auto” indicate when participants just watched the screen with no instructions to mimic images of facial expressions. Blocks labeled “Effort” indicate when participants were told to actively mimic images of facial expressions.

After viewing the three 10-image blocks testing for automatic mimicry, participants were tested on effortful mimicry. They were shown the same images in the same order and using the same timings as the previous automatic mimicry blocks. We also used the same between block rest procedures as above. The major difference was that this time, they were also asked to purposefully mimic the facial expressions while these effortful facial muscle changes were measured. Specifically, participants viewed instructions on the computer monitor to “make an expression just like the one on the screen,” during the ITI period. Participants were asked to mimic the expressions for each photo for the entire block of trials. They were not required to continue to produce the facial expression between photos of different facial expressions (during the ITI period). After all 30 images were viewed twice and both automatic and effortful mimicry were assessed, participants were debriefed. Please refer to Figure 3 for a depiction of the procedural steps.

#### Manipulation checks

Manipulation checks were conducted with paired samples *t*-tests to determine whether average facial muscle activation at baseline was significantly different from average facial muscle activation in the 4-s period following the presentation of each image in each set (see Table 2 for detailed statistical results of these tests). As expected, significant increases were found in Zygo and Orbic activation from before to after the presentation of happy images in automatic mimicry, with similar significant increases in Corr activation from before to after the presentation of sad images in automatic mimicry. Similarly, in the effortful conditions, significant increases were found in Zygo and Orbic from before to after the presentation of happy images, as were significant increases in Corr after the presentation of sad images.

**Table 2:** Differences between average baseline facial muscle activation and average activation 4 S post-image presentation in response to happy, sad, and neutral faces.

Variable		Mean	*t*	*p* (2-tailed)
Automatic happy				
Corr	Baseline	0.51	2.40	0.018
	Post-image	0.56		
Orbic	Baseline	0.24	5.59	<0.001
	Post-image	0.36		
Zygo	Baseline	0.15	3.41	<0.001
	Post-image	0.22		
Automatic sad				
Corr	Baseline	0.51	4.11	<0.001
	Post-image	0.62		
Orbic	Baseline	0.28	3.47	<0.001
	Post-image	0.35		
Zygo	Baseline	0.17	1.32	0.190
	Post-image	0.19		
Automatic neutral				
Corr	Baseline	0.51	5.31	<0.001
	Post-Image	0.61		
Orbic	Baseline	0.23	2.37	0.019
	Post-Image	0.27		
Zygo	Baseline	0.15	1.29	0.200
	Post-Image	0.17		
Effortful happy				
Corr	Baseline	0.52	−4.44	<0.001
	Post-Image	0.41		
Orbic	Baseline	0.26	21.62	<0.001
	Post-Image	0.89		
Zygo	Baseline	0.17	19.70	0.095
	Post-Image	0.86		
Effortful sad				
Corr	Baseline	0.52	17.25	<0.001
	Post-image	1.08		
Orbic	Baseline	0.25	11.04	0.003
	Post-image	0.52		
Zygo	Baseline	0.18	6.59	<0.001
	Post-image	0.29		
Effortful neutral				
Corr	Baseline	0.51	4.12	<0.001
	Post-image	0.62		
Orbic	Baseline	0.22	5.96	<0.001
	Post-image	0.33		
Zygo	Baseline	0.17	3.68	<0.001
	Post-image	0.23		

Corr = Corrugator Supercilii; Orbic = Orbicularis Oculi; Zygo = Zygomaticus Major.

#### Analytical procedure

First, bivariate correlations were conducted between variables of interest and the age, sex, and race of participants to determine needed covariates. Based on preliminary analyses, only age was needed as a covariate (specifically in analyses with NE and loneliness) and was thus added to these regression models.

Multiple regression was then used to answer the following questions:^1^ (a) Is there an association between NM (the independent variable [IV]; assessed via multiple measures) and the ability to automatically and effortfully mimic others’ facial expressions? (dependent variables [DVs] were the specific muscle group change scores from baseline described in the EMG section) and (b) Is greater facial muscle activation change from baseline during automatic and/or effortful mimicry (DVs) associated with greater social support and less loneliness (IVs, tested independently) across all participants (and vice versa for incorrect or no activation)? Across analyses, the alpha level was set at *p* < 0.05.

#### Preliminary analyses

Because nine individuals in the current sample reported the diagnosis of a psychological disorder, ANOVA was used to examine differences between those with and without a psychological disorder. Of these nine individuals, only two indicated a current diagnosis of clinical depression. Results revealed significantly greater BDI-II scores for those with a psychological disorder (*M* = 16.56) than without (*M* = 7.06), *F*(1,133) = 12.81, *p* < 0.001, as well as higher baseline trait NM scores for those with a psychological disorder (*M* = 1.76) than without (*M* = 1.13), *F*(1, 133) = 7.46, *p* = 0.007. Individuals reporting a psychological disorder were not excluded from analyses, as this subset theoretically represents an important part of the continuum of NM of interest in the current set of analyses (i.e., the high end of NM).^2^

## Results

### Is there an association between negative mood and the ability to automatically and effortfully mimic others’ facial expressions?

For a summary of the following results, please see Table 3.

**Table 3:** Standardized ß values from significant and marginal regression analyses examining the relationship between negative mood and facial mimicry.

Variable	NM	NE	BDI	DM	DE
Automatic happy					
Corr	NS	NS	NS	NS	NS
Orbic	NS	NS	NS	NS	NS
Zygo	NS	−0.16^†^	NS	NS	−0.20*
Automatic neutral					
Corr	NS	NS	NS	NS	NS
Orbic	NS	NS	NS	NS	NS
Zygo	NS	NS	NS	NS	NS
Automatic sad					
Corr	NS	NS	NS	NS	NS
Orbic	NS	NS	NS	NS	NS
Zygo	NS	NS	NS	NS	NS
Effortful happy					
Corr	NS	NS	NS	NS	NS
Orbic	NS	−0.20*	NS	−0.19*	−0.18*
Zygo	NS	NS	−0.19*	NS	−0.17^†^
Effortful neutral					
Corr	NS	NS	NS	NS	NS
Orbic	0.18^†^	NS	NS	NS	NS
Zygo	NS	NS	NS	NS	NS
Effortful sad					
Corr	NS	NS	NS	NS	NS
Orbic	NS	NS	NS	NS	NS
Zygo	0.16^†^	NS	0.16^†^	0.19*	0.22*

Corr = Corrugator Supercilii; Orbic = Orbicularis Oculi; Zygo = Zygomaticus Major. Age was controlled for throughout analyses based on associations with relevant outcomes. **p < 0.01; *p < 0.05; ^†^*p* ≤ 0.10.

#### Mimicry of happy faces

Automatic Mimicry: High NE was marginally statistically associated with less change in Zygo muscle activation in automatic response to happy facial expressions (ß = −0.16, *p* < 0.10; *R*^2^ = 0.03, *F*(1, 124) = 3.37, *p* = 0.069). Furthermore, a statistically significant relationship was found between high levels of DE and less change in Zygo muscle activation following happy facial expressions (ß = −0.20, *p* < 0.05, *R*^2^ = 0.04, *F*(1, 122) = 4.99, *p* = 0.027). No comparable associations were found for general NM, DM, or depressive symptoms and Zygo muscle activation, as was the case for associations between any of the measures of NM and Orbic or Corr muscle activation.

**Figure 4:**
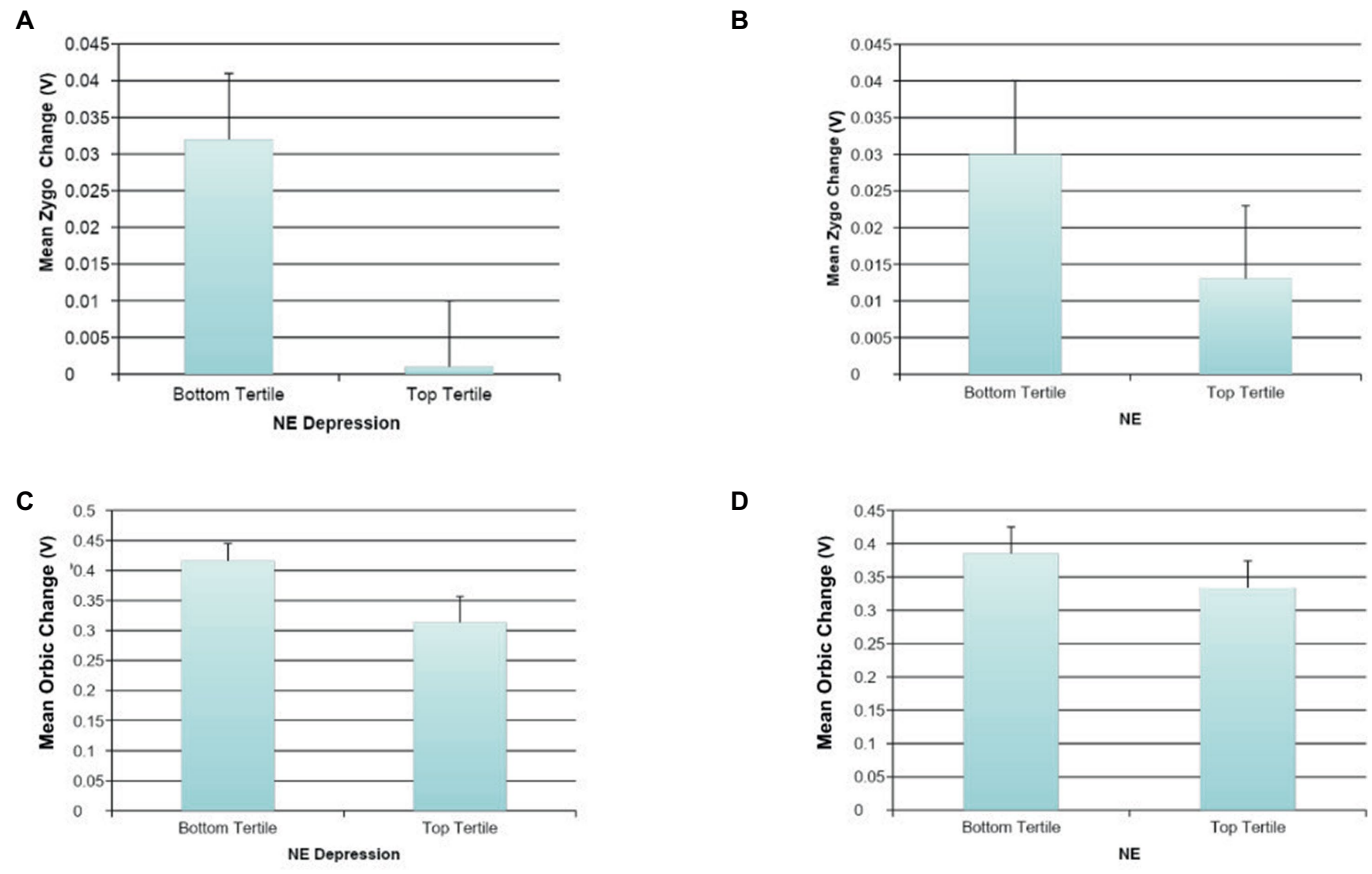
The association between negative mood and mimicry of happy faces. **(A)** The association between NE depression and automatic happy faces mimicry for Zygomaticus Major (Zygo). **(B)** The association between NE and automatic happy faces mimicry for Zygomaticus Major (Zygo). **(C)** The association between NE depression and effortful happy faces mimicry for Orbicularis Oculi (Orbic). **(D)** The association between NE and effortful happy faces mimicry for Orbicularis Oculi (Orbic).

Effortful Mimicry: Greater change in Zygo activation was associated with fewer depressive symptoms (ß = −0.19, *p* < 0.05, *R*^2^ = 0.04, *F*(1, 123) = 4.59, *p* = 0.034) and marginally with lower levels of DE (ß = −0.17, *p* < 0.10, *R*^2^ = 0.03, *F*(1, 122) = 3.41, *p* = 0.067). There were no statistically significant associations between Zygo muscle activation and NE, general NM, or DM. Greater change in Orbic activation was statistically significantly associated with less general NE (ß = −0.20, *p* < 0.05, *R*^2^ = 0.08, *F*(2, 122) = 5.00, *p* = 0.008); lower levels of DM (ß = −0.19, *p* < 0.05 *R*^2^ = 0.04, *F*(1, 123) = 4.38, *p* = 0.038); and lower levels of DE (ß = −0.18, *p* < 0.05, *R*^2^ = 0.03, *F*(1, 122) = 3.96, *p* = 0.049). Orbic muscle activation and general NM or depressive symptoms were not associated at significant levels nor were there associations between Corr muscle activation and any of the measures of NM. See Figure 4 for graphs of the more interesting findings.

#### Mimicry of neutral faces

Automatic Mimicry: As expected, no statistically significant associations were found between automatic change in muscle activation following the presentation of neutral facial expressions and NM.

Effortful Mimicry: There were no statistically significant associations between Zygo muscle activation and any of the measures of NM. For Orbic muscle activation, the only finding that was approaching significance was that greater change in Orbic activity was associated with greater general NM (ß = 0.18, *p* < 0.10, *R*^2^ = 0.03, *F*(1, 121) = 3.79, *p* = 0.054), indicating that those higher in general NM were incorrectly activating muscles around their eyes when intentionally mimicking a neutral face. There were no other associations with Orbic or Corr and any of the measures of NM.

#### Mimicry of sad faces

Automatic Mimicry: As expected, no statistically significant associations were found between automatic change in muscle activation following the presentation of sad facial expressions and NM.

**Figure 5:**
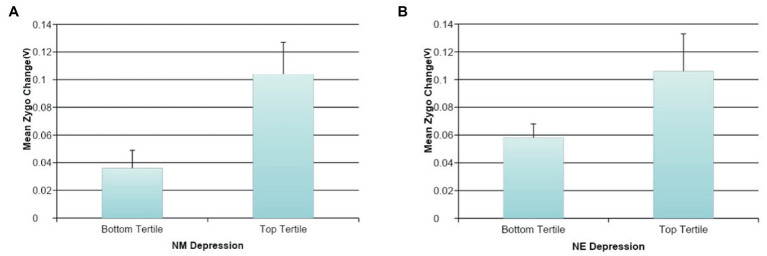
The association between negative mood and mimicry of sad faces. **(A)** The association between NM depression and effortful sad faces mimicry for Zygomaticus Major (Zygo). **(B)** The association between NE depression and effortful sad faces mimicry for Zygomaticus Major (Zygo).

Effortful Mimicry: Contrary to our initial hypotheses, greater effortful change in Zygo activity following the presentation of sad facial expressions was found to be statistically significantly associated with higher levels of DM (ß = 0.19, *p* < 0.05, *R*^2^ = 0.04, *F*(1, 123) = 4.59, *p* = 0.034); higher levels of DE (ß = 0.22, *p* < 0.05, *R*^2^ = 0.05, *F*(1, 123) = 6.15, *p* = 0.014); higher general NM (ß = 0.16, *p* < 0.10, *R*^2^ = 0.03, *F*(1, 124) = 3.29, *p* = 0.072); and depressive symptoms (ß = 0.16, *p* < 0.10, *R*^2^ = 0.02, *F*(1, 124) = 3.05, *p* = 0.083). This indicates that when trying to mimic sadness, individuals higher in measures of depressed/dysphoric mood (but not all types of NM) were activating the *incorrect* facial muscle group, the Zygo muscle in the cheek. There were no statistically significant associations between Zygo muscle activation and NE or DM. There were also no statistically significant associations between Orbic or Corr muscle activation and any of the measures of NM. See Figure 5 for graphs of the more interesting findings.

### Is there an association between facial muscle activation change from baseline during automatic and effortful facial expression mimicry and social functioning?

#### Mimicry of happy faces

Automatic Mimicry: There were no statistically significant associations between facial muscle activation and social functioning during automatic mimicry.

**Figure 6:**
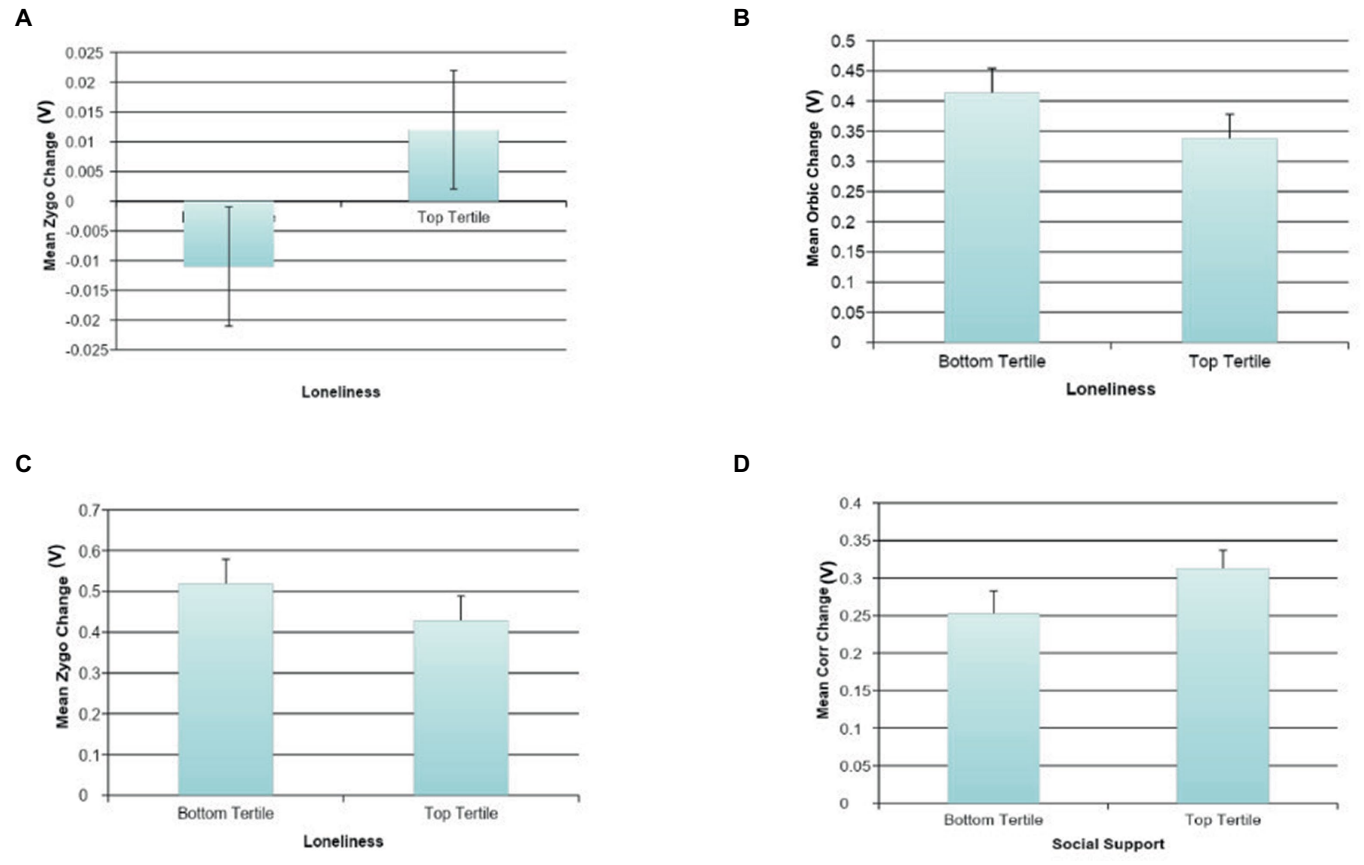
The association between social variables and facial mimicry. **(A)** The association between loneliness and automatic sad face mimicry for Zygomaticus Major (Zygo). **(B)** The association between loneliness and effortful happy face mimicry for Orbicularis Oculi (Orbic). **(C)** The association between loneliness and effortful happy face mimicry for Zygomaticus Major (Zygo). **(D)** The association between social support and effortful sad face mimicry for Corrugator (Corr).

Effortful Mimicry: Higher loneliness was associated with less Orbic activation change (ß = −0.15, *p* < 0.05, *R*^2^ = 0.05, *F*(2, 122) = 3.43, *p* = 0.036), and less Zygo activation change (ß = −0.15, *p* < 0.05, *R*^2^ = 0.06, *F*(2, 122) = 3.51, *p* = 0.033) after happy image presentation (Figures 6B,C). This suggests less activity in the correct muscle groups following the presentation of happy facial expressions in those with greater loneliness. Similar associations were not found between Corr muscle activation and social functioning.

#### Mimicry of neutral faces

There were no statistically significant associations between facial muscle activation and social functioning during either automatic or effortful mimicry.

#### Mimicry of sad faces

Automatic Mimicry: Greater automatic change in Zygo activation following the presentation of sad facial expressions was associated with self-reported loneliness (ß = 0.17, *p* < 0.10, *R*^2^ = 0.07, *F*(2, 125) = 4.35, *p* = 0.015; Figure 6A), which indicates that automatically activating the incorrect muscle group following the presentation of sad facial expressions is associated with higher loneliness. There were no statistically significant associations between Orbic or Corr muscle activation and social functioning.

Effortful Mimicry: Following the presentation of sad facial expressions in the effortful condition, a statistically significant association was found between higher self-reported social support and greater Corr activation change (ß = 0.21, *p* < 0.05, *R*^2^ = 0.04, *F*(1, 124) = 5.60, *p* = 0.019) (Figure 6D). There was also a marginal association between higher self-reported social support and less Zygo activation change (ß = −0.17, *p* < 0.10, *R*^2^ = 0.03, *F*(1, 124) = 3.81, *p* = 0.053). This suggests that those with high social support are more skilled at intentionally demonstrating sadness. This finding was replicated in the loneliness data such that those with lower loneliness showed higher Corr activation change (ß = −0.17, *p* < 0.05, *R*^2^ = 0.07, *F*(2, 123) = 4.62, *p* = 0.012), as well as less Zygo activation change (ß = 0.21, *p* < 0.05, *R*^2^ = 0.08, *F*(2, 123) = 5.51, *p* = 0.005). See Figure 6 for graphs of the more interesting findings.

## Discussion

The current study examined associations between transient NM and automatic and effortful facial expression mimicry. Although many studies have reported deficits in automatic mimicry for individuals with depression and dysphoria (Wexler et al., 1993; Sloan et al., 2002; Lautzenhiser, 2003), this study is the first to also examine both automatic and effortful facial expression mimicry in the context of transient negative moods and emotion and in a healthy sample. Results support previous clinical depression and dysphoria findings by verifying some associations between NM and less change in facial muscle activation during automatic mimicry of positive facial expressions (in our findings, accounting for approximately 3–4% of the variability in automatic Zygo mimicry of positive expression). More importantly, results add to previous literature by confirming a significant association between NM and less change in facial muscle activation during *effortful* mimicry of positive facial expressions. Specifically, negative moods and even transient negative emotions (especially depressed ones) accounted for up to 8% of the variability in Orbic mimicry of positive expressions. These findings confirm the central hypothesis that even mild negative moods in healthy samples are tied with detriments to mimicry ability, especially for positive emotion.

Also consistent with past research on depression and automatic mimicry, there were no associations between automatic mimicry of negative and neutral expressions in those with higher NM. It is worthwhile to note that automatic facial expressions are typically associated with positive expressions (e.g., happiness), while negative expressions (e.g., sadness, anger, disgust) are mimicked less often and with less intensity (Bourgeois and Hess, 2008; Fischer and Hess, 2017). When presented with a positive expression, people are more compelled to return the pleasant expression in order to facilitate a smooth and enjoyable interaction, whereas being confronted with a negative expression decreases the desire to invest in the social interaction and, therefore, leads to less mimicry (Likowski et al., 2008). Thus, it can be assumed that facial mimicry relates more to affiliative emotions, since positive expressions are more of an affiliative signal than negative ones (Hess, 2021; Hess and Fischer, 2022). This might explain the lack of significant associations between automatic mimicry of negative and neutral expressions and high NM.

Interestingly, new findings were revealed in the effortful mimicry condition such that individuals reporting high levels of state negative affect, especially self-reported depressed feelings, also have impaired effortful mimicry of *sad* facial expressions, with increased activation of incorrect muscle groups (i.e., the zygomaticus major muscle) occurring, a muscle group involved in smiling, contentment, and pleasure (Ekman and Friesen, 1978). This association between negative moods/emotions and Zygo activation to sadness ranged from approximately 2–5% of the variance explained. Previous literature on depressed individuals has shown deficits in automatic mimicry of positive expressions, with greater Corr activation, which is typically not associated with positive expressions (Sloan et al., 2002); however, activation of incorrect muscle groups following intentional mimicry of sad facial expressions has not been seen previously. This could have significant practical implications in social situations for sad and/or dysphoric individuals if they are unable to accurately mimic others’ expressions, and especially if they inappropriately smile in the face of others’ sadness, as was seen here.

These deficits seen in effortful mimicry of facial expressions for individuals reporting elevated NM could have implications for clinical treatments for individuals with mild to moderate depression. To date, social skills training (SST), which often includes instruction and coaching related to effective non-verbal communication, has been widely employed in conjunction with other therapies to treat symptoms of depression and enhance social functioning in this population (see Segrin, 2000). Although SST has generally been found to be an effective adjunctive treatment for many individuals with depression (Segrin, 2000), the current results suggest that basic awareness and instruction in facial expression mimicry may not alone be effective in helping depressed individuals accurately mimic facial expressions. Additionally, it has been well documented that individuals with depression process facial expressions differently than non-depressed individuals, showing deficiencies in the ability to process expressions effortfully. This particularly affects processing of positive expressions (Hartlage et al., 1993), as well as leads to a bias in perceiving negative facial expressions, which can serve to maintain a depressive view of one’s social environment (Bistricky et al., 2011). Results of the current study suggest that this processing deficiency may also be present in those with subclinical levels of depressive symptoms and that simply asking dysphoric individuals to purposefully mimic a positive expression will not lead to successful mimicry. Future studies should examine whether a more explicit SST module, with instruction about the specific muscle groups involved in positive facial expression (i.e., the Orbic and Zygo muscles), could lead to more accurate effortful mimicry of positive facial expressions.

Because of the large literature on the importance of facial expressions in social interactions (Izard, 1989; Manstead, 1991; Keltner and Haidt, 1999), we hypothesized that greater facial muscle activation change from baseline during both automatic and effortful mimicry (i.e., correct muscle activation and clearer mimicry) would be associated with higher levels of social support and less loneliness. Our hypothesis was confirmed; across mimicry conditions, clear associations were found between activation of the correct muscle group for the expression at hand and both greater social support and less loneliness. A significant association was found between activation of the incorrect Zygo muscle during both automatic and effortful mimicry of sad facial expressions and greater self-reported loneliness; however, no significant results were found between social variables and automatic mimicry of happy and neutral faces. The presence of Zygo activity during effortful mimicry of sad faces was also negatively associated with social support. The presence of Corr activity, which is appropriate during mimicry of sad facial expressions, was *positively* associated with social support and *negatively* associated with loneliness, which could indicate that activation of the correct muscle group for the expression leads to better social functioning (or vice versa). This is also supported by results showing that greater Orbic and Zygo activity during effortful happy facial expressions, which is appropriate for the expression, was associated with decreased loneliness.

Taken together, these social results point to the previously discussed studies citing the importance of accurate facial mimicry for healthy social functioning (Tuck et al., 2016a). Studies have repeatedly suggested that isolation and loneliness contribute to depressive symptoms (Rockwell et al., 1976; Palinkas and Browner, 1995; Cacioppo et al., 2010). Further, it can be argued that the relationship between poor social interactions and negative affect is bidirectional, with depression leading to greater social withdrawal and less confidence in social ability, and alternatively, perceived or actual social difficulties leading to increased depression (Bistricky et al., 2011). However, the current study also points to the intriguing possibility that improving facial mimicry skill could contribute to the discontinuation of this cycle by leading to more effective and rewarding social experiences for individuals with dysphoria and depression. In line with this, greater ability to express a certain emotion in the face when asked to do so has been found to be associated with fewer depressive symptoms and better health (Tuck et al., 2016a).

The current study does have limitations to consider, such as limited generalizability to non-clinical, White student samples. Future research should seek to examine whether this replicates in more diverse samples. Also, as mood was naturally occurring in these studies, our understanding of directionality is limited, necessitating future work using emotion inductions to better understand causal directionality as well as whether brief, induced negative emotional states have the same mimicry effect. An additional limitation in the interpretation of these findings is the overtness of the stimuli. Some facial researchers hypothesize that mimicry is more useful in subtle expression contexts (Dimberg et al., 2000; Prochazkova and Kret, 2017); thus, the meaning of these findings with overt stimuli cannot generalize to those situations and the results might have been different with alternative stimuli. It is also relevant to note that this study used static images rather than videos (Cohen et al., 2003), which can strengthen ecological validity in terms of how individuals respond to expressions in the real world. Finally, because of the exploratory nature of the findings (we did not know which types of negative affect would be important nor whether findings from the clinical literature would apply with transient mood) and the necessary large number of analyses that go with this, we did not use corrected *p*-values. We hope that the results from this study will guide more targeted studies in the future to determine the replicability of the findings.

Despite limitations of the current work, results have contributed to filling an important gap in the literature by examining the association between NM and effortful facial expression mimicry. Understanding whether deficits in effortful mimicry exist could fundamentally change recommended treatments for depression, particularly those that include a SST component. Given the estimation that millions of people experience dysphoria at some period in their lives (Fromson and Bell, 2010), not to mention the regular day to day negative moods we all experience, the current results could potentially benefit a large range of individuals by pointing to the commonness of this mimicry difficulty with high NM. Finally, these findings highlight the need to control for baseline mood in experimental facial manipulation studies. For example, facial feedback work where individuals are asked to pose their face in a certain manner (Coles et al., 2019) might have large amounts of noise based on the current affective state of participants. Future facial mimicry and expression studies may need to consider controlling mood or using a neutral mood induction prior to manipulations to ensure equal expression ability across all participants.

## Data availability statement

The original contributions presented in the study are included in the article, further inquiries can be directed to the corresponding author.

## Ethics statement

The studies involving human participants were reviewed and approved by University of Kansas IRB. The patients/participants provided their written informed consent to participate in this study. Written informed consent was obtained from the individual(s) for the publication of any potentially identifiable images or data included in this article.

## Author contributions

TK-F developed the study concept. SP and RI contributed to the study design. Testing and data collection were performed by TK-F and CG. TK-F performed the data analysis and interpretation under the supervision of SP and RI. TK-F and MC contributed to the writing of the manuscript at various stages. SP, JL, MC, and CG provided critical revisions. All authors contributed to the article and approved the submitted version.

## Conflict of interest

The authors declare that the research was conducted in the absence of any commercial or financial relationships that could be construed as a potential conflict of interest.

## Publisher’s note

All claims expressed in this article are solely those of the authors and do not necessarily represent those of their affiliated organizations, or those of the publisher, the editors and the reviewers. Any product that may be evaluated in this article, or claim that may be made by its manufacturer, is not guaranteed or endorsed by the publisher.

## Footnotes

^1^There has been much debate over familywise alpha and a gold-standard method to correct for multiple comparisons. Some argue for the importance of familywise alpha tests like false discovery rate and Bonferroni correction (see Matsunaga, 2007), while others argue these reduce power among other problems, like increased Type II error, ambiguity as to what constitutes a family, etc. (see Feise, 2002; O’Keefe, 2003). Given this debate over familywise alpha corrections, as well as the ambiguity of what constitutes a family in our study, we did not use Bonferroni correction or false discovery rate. Also, since this is an exploratory study, we did not lower the alpha level. We believe the effect sizes (R-squared change) are more meaningful than strict statistical significance in our study. As such, we focus on the strengths of our effects in the interpretation of our findings.

^2^Analyses were also run removing the nine individuals diagnosed with a psychological disorder and no notable changes in results were found.

## References

[ref1] Arnold A. Winkielman P. (2021). Smile (but only deliberately) though your heart is aching: loneliness is associated with impaired spontaneous smile mimicry. Soc. Neurosci. 16, 26–38. doi: 10.1080/17470919.2020.1809516, PMID: 32835612

[ref2] Bargh J. A. Williams E. L. (2006). The automaticity of social life. Curr. Dir. Psychol. Sci. 15, 1–4. doi: 10.1111/j.0963-7214.2006.00395.x, PMID: 18568084 PMC2435044

[ref4] Beck A. T. Steer R. A. Brown G. K. (1996). Manual for the Beck Depression Inventory – II. San Antonio, TX: Psychological Corporation.

[ref5] Bistricky S. L. Ingram R. E. Atchley R. A. (2011). Facial affect processing and depression susceptibility: cognitive biases and cognitive neuroscience. Psychol. Bull. 137, 998–1028. doi: 10.1037/a0025348, PMID: 21895353

[ref6] Bourgeois P. Hess U. (2008). The impact of social context on mimicry. Biol. Psychol. 77, 343–352. doi: 10.1016/j.biopsycho.2007.11.008, PMID: 18164534

[ref9] Brothers L. (1990). The neural basis of primate social communication. Motiv. Emot. 14, 81–91. doi: 10.1007/BF00991637

[ref10] Cacioppo J. T. Hawkley L. C. Thisted R. A. (2010). Perceived social isolation makes me sad: 5-year cross-lagged analyses of loneliness and depressive symptomatology in the Chicago health, aging, and social relations study. Psychol. Aging 25, 453–463. doi: 10.1037/a0017216, PMID: 20545429 PMC2922929

[ref11] Cacioppo J. T. Petty R. E. Losch M. E. Kim H. S. (2008). Electromyographic Activity Over Facial Muscle Regions Can Differentiate the Valence and Intensity of Affective Reactions. (pp. 69–83). New York, NY, US: Psychology Press. 10.1037//0022-3514.50.2.260 3701577

[ref12] Cappella J. N. (1993). The facial feedback hypothesis in human interaction: review and speculation. J. Lang. Soc. Psychol. 12, 13–29. doi: 10.1177/0261927X93121002

[ref13] Chartrand T. L. Bargh J. A. (1999). The chameleon effect: the perception-behavior link and social interaction. J. Pers. Soc. Psychol. 76, 893–910. doi: 10.1037//0022-3514.76.6.893, PMID: 10402679

[ref15] Cohen S. Hoberman H. M. (1983). Positive events and social supports as buffers of life change stress. J. Appl. Soc. Psychol. 13, 99–125. doi: 10.1111/j.1559-1816.1983.tb02325.x

[ref16] Cohen S. Mermelstein R. Kamarck T. Hoberman H. M. (1985). “Measuring the functional components of social support” in Social Support: Theory, Research and Applications eds. Sarason I. G. Sarason B. R., (Netherlands: Springer), 73–94.

[ref01] Cohen I. Sebe N. Garg A. Chen L. S. Huang T. S. (2003). Facial expression recognition from video sequences: Temporal and static modeling. Comput. Vis. Image Underst. 91, 160–187. doi: 10.1016/S1077-3142(03)00081-X

[ref17] Coles N. A. Larsen J. T. Lench H. C. (2019). A meta-analysis of the facial feedback literature: effects of facial feedback on emotional experience are small and variable. Psychol. Bull. 145, 610–651. doi: 10.1037/bul0000194 30973236

[ref18] Coles N. March D. S. Marmolejo-Ramos F. Larsen J. T. Arinze N. C. Ndukaihe I. L. G.. (2022). A multi-lab test of the facial feedback hypothesis by the many smiles collaboration. Nat. Hum. Behav. 6, 1731–1742. doi: 10.1038/s41562-022-01458-9, PMID: 36266452

[ref19] Cozolino L. (2002). The Neuroscience of Psychotherapy. New York, NY: Norton.

[ref21] Darwin C. R. (1965). The Expression of Emotions in Man and Animals. Chicago: University of Chicago Press.

[ref22] Dimberg U. (1982). Facial reactions to facial expressions. Psychophysiology 19, 643–647. doi: 10.1111/j.1469-8986.1982.tb02516.x 7178381

[ref02] Dimberg U. (1990). Facial electromyography and emotional reactions. Psychophysiology 27, 481–494. 2274612 10.1111/j.1469-8986.1990.tb01962.x

[ref23] Dimberg U. Thunberg M. (1998). Rapid facial reactions to emotional facial expressions. Scand. J. Psychol. 39, 39–45. doi: 10.1111/1467-9450.00054 9619131

[ref24] Dimberg U. Thunberg M. Elmehed K. (2000). Unconscious facial reactions to emotional facial expressions. Psychol. Sci. 11, 86–89. doi: 10.1111/1467-9280.00221 11228851

[ref25] Dimberg U. Thunberg M. Grunedal S. (2002). Facial reactions to emotional stimuli: automatically controlled emotional responses. Cognit. Emot. 16, 449–471. doi: 10.1080/02699930143000356

[ref26] Dozois D. J. A. Dobson K. S. Ahnberg J. L. (1998). A psychometric evaluation of the Beck depression inventory–II. Psychol. Assess. 10, 83–89. doi: 10.1037/1040-3590.10.2.83

[ref27] Duchenne G. B. (1990). The Mechanism of Human Facial Expression. (R. A. Cuthbertson, Ed. and Trans.). Cambridge: Cambridge University Press.

[ref28] Duffy K. A. Chartrand T. L. (2015). Mimicry: causes and consequences. Curr Opin Behav Sci 3, 112–116. doi: 10.1016/j.cobeha.2015.03.002

[ref30] Ekman P. (1992). An argument for basic emotions. Cognit. Emot. 6, 169–200. doi: 10.1080/02699939208411068

[ref31] Ekman P. Friesen W. V. (1978). Manual of Facial Action Coding System (FACS). Palo Alto, CA: Consulting Psychologists Press.

[ref32] Ekman P. Friesen W. V. (1982). Felt, false, and miserable smiles. J. Nonverbal Behav. 6, 238–252. doi: 10.1007/BF00987191

[ref33] Feise R. J. (2002). Do multiple outcome measures require p-value adjustment? BMC Med. Res. Methodol. 2:8. doi: 10.1186/1471-2288-2-8, PMID: 12069695 PMC117123

[ref34] Field T. M. Woodson R. Greenberg R. Cohen D. (1982). Discrimination and imitation of facial expressions by neonates. Science 218, 179–181. doi: 10.1126/science.7123230, PMID: 7123230

[ref35] Fischer A. Hess U. (2017). “Mimicking emotions,” in Current Opinion in Psychology. Elsevier B.V, 151–155. 10.1016/j.copsyc.2017.07.008 28950963

[ref36] Forbes P. A. G. Pan X. Antonia A. F. (2016). Reduced mimicry to virtual reality avatars in autism Spectrum disorder. J. Autism Dev. Disord. 46, 3788–3797. doi: 10.1007/s10803-016-2930-2, PMID: 27696183 PMC5110595

[ref37] Fridlund A. J. Cacioppo J. T. (1986). Guidelines for human electromyographic research. Psychophysiology 23, 567–589. doi: 10.1111/j.1469-8986.1986.tb00676.x 3809364

[ref03] Fromson J. A. Bell I. R. (2010). “Depression,” in Decision Making in Medicine: An Algorithmic Approach. eds. Muslin S. B. Greene II H. L., 3rd ed. (Mosby), 662–665.

[ref39] Goeleven E. De Raedt R. Leyman L. Verschuere B. (2008). The Karolinska directed emotional faces: a validation study. Cognit. Emot. 22, 1094–1118. doi: 10.1080/02699930701626582

[ref40] Goldman A. I. Sripada C. S. (2005). Simulationist models of face-based emotion recognition. Cognition 94, 193–213. doi: 10.1016/j.cognition.2004.01.005, PMID: 15617671

[ref42] Grove J. R. Prapavessis H. (1992). Preliminary evidence for the reliability and validity of an abbreviated profile of mood states. Int. J. Sport Psychol. 23, 93–109.

[ref44] Hartlage S. Alloy L. B. Vazquez C. Dykman B. (1993). Automatic and effortful processing in depression. Psychol. Bull. 113, 247–278. doi: 10.1037/0033-2909.113.2.247 8451334

[ref45] Hatfield E. Bensman L. Thornton P. D. Rapson R. L. (2014). New perspectives on emotional contagion: a review of classic and recent research on facial mimicry and contagion. Interpersona Int J Pers Relationsh 8, 159–179. doi: 10.5964/ijpr.v8i2.162

[ref46] Hatfield E. Cacioppo J. T. Rapson R. L. (1992). “Primitive emotional contagion” in Emotion and Social Behavior. ed. Clark M. S. (Thousands Oaks, CA, US: Sage Publications, Inc), 151–177.

[ref04] Hays R. D. Dimatteo M. R. (1987). A Short-Form Measure of Loneliness. J. Pers. Assess. 51, 69–81. doi: 10.1207/s15327752jpa5101_6 3572711

[ref47] Hess U. (2021). Who to whom and why: the social nature of emotional mimicry. Psychophysiology 58:e13675. doi: 10.1111/psyp.13675, PMID: 32915999

[ref48] Hess U. Blairy S. (2001). Facial mimicry and emotional contagion to dynamic emotional facial expressions and their influence on decoding accuracy. Int. J. Psychophysiol. 40, 129–141. doi: 10.1016/S0167-8760(00)00161-6 11165351

[ref49] Hess U. Fischer A. (2022). Emotional mimicry as social regulator: theoretical considerations. Cognit. Emot. 36, 785–793. doi: 10.1080/02699931.2022.2103522, PMID: 35920780

[ref50] Ingram R. E. Hamilton N. A. (1999). Evaluating precision in the social psychological assessment of depression: methodological considerations, issues, and recommendations. J. Soc. Clin. Psychol. 18, 160–180. doi: 10.1521/jscp.1999.18.2.160

[ref51] Izard C. E. (1989). The Structure and Functions of Emotions: Implications for Cognition, Motivation, and Personality. (pp. 39–73). Washington, DC, US: American Psychological Association.

[ref53] Jenkins B. N. Cross M. P. Donaldson C. D. Pressman S. D. Fortier M. A. Kain Z. N.. (2021). The subcomponents of affect scale (SAS): validating a widely used affect scale. Psychol. Health, 1–19. doi: 10.1080/08870446.2021.2000612, PMID: 34846253

[ref54] Kang J. Derva D. Kwon D. Y. Wallraven C. (2019). Voluntary and spontaneous facial mimicry toward other’s emotional expression in patients with Parkinson’s disease. PLoS One 14:e0214957. doi: 10.1371/journal.pone.0214957, PMID: 30973893 PMC6459535

[ref55] Keltner D. Haidt J. (1999). Social functions of emotions at four levels of analysis. Cognit. Emot. 13, 505–521. doi: 10.1080/026999399379168

[ref59] Krumhuber E. G. Likowski K. U. Weyers P. (2014). Facial mimicry of spontaneous and deliberate Duchenne and non-Duchenne smiles. J. Nonverbal Behav. 38, 1–11. doi: 10.1007/s10919-013-0167-8

[ref60] Lanzetta J. T. Englis B. G. (1989). Expectations of cooperation and competition and their effects on observers' vicarious emotional responses. J. Pers. Soc. Psychol. 56, 543–554. doi: 10.1037/0022-3514.56.4.543

[ref61] Lautzenhiser L. M. (2003). Affective responses in normal and depressed children. Dissertation Abstracts International: Section B: The Sciences and Engineering. ProQuest Information & Learning, US. Retrieved from http://resources.library.brandeis.edu/login?url=http://search.ebscohost.com/login.aspx?direct=true&db=psyh&AN=2003-95016-008&site=ehost-live&scope=site

[ref63] Lewinsohn P. M. (1974). A Behavioral Approach to Depression. (pp. 318–xvii, 318). Oxford, England: John Wiley & Sons, Oxford.

[ref05] Lewis M. B. Dunn E. (2017). Instructions to mimic improve facial emotion recognition in people with sub-clinical autism traits. Q. J. Exp. Psychol. 70, 2357–2370. doi: 10.1080/17470218.2016.1238950 27734764

[ref64] Likowski K. U. Mühlberger A. Seibt B. Pauli P. Weyers P. (2008). Modulation of facial mimicry by attitudes. J. Exp. Soc. Psychol. 44, 1065–1072. doi: 10.1016/j.jesp.2007.10.007

[ref65] Lischetzke T. Cugialy M. Apt T. Eid M. Niedeggen M. (2020). Are those who tend to mimic facial expressions especially vulnerable to emotional contagion? J. Nonverbal Behav. 44, 133–152. doi: 10.1007/s10919-019-00316-z

[ref66] Lishner D. A. Cooter A. B. Zald D. H. (2008). Rapid emotional contagion and expressive congruence under strong test conditions. J. Nonverbal Behav. 32, 225–239. doi: 10.1007/s10919-008-0053-y

[ref06] Lundqvist L. O. Dimberg U. (1995). Facial expressions are contagious. J. Psychophysiol. 9, 203–211.

[ref67] Lundqvist D. Flykt A. Ohman A. (1998). The Karolinska Directed Emotional Faces (KDEF). Stockholm: Department of Neurosciences Karolinska Hospital.

[ref68] Manstead A. S. (1991). Emotion in social life. Cognit. Emot. 5, 353–362. doi: 10.1080/02699939108411047

[ref69] Matsunaga M. (2007). Familywise error in multiple comparisons: disentangling a knot through a critique of O’Keefe’s arguments against alpha adjustment. Commun. Methods Meas. 1, 243–265. doi: 10.1080/19312450701641409

[ref70] McIntosh D. N. Reichmann-Decker A. Winkielman P. Wilbarger J. L. (2006). When the social mirror breaks: deficits in automatic, but not voluntary, mimicry of emotional facial expressions in autism. Dev. Sci. 9, 295–302. doi: 10.1111/j.1467-7687.2006.00492.x, PMID: 16669800

[ref71] McNair D. M. Lorr M. Droppleman L. F. (1971). Manual for the Profile of Mood States. San Diego, CA: Educational and Industrial Testing Services.

[ref73] Moody E. J. McIntosh D. N. Mann L. J. Weisser K. R. (2007). More than mere mimicry? The influence of emotion on rapid facial reactions to faces. Emotion 7, 447–457. doi: 10.1037/1528-3542.7.2.447, PMID: 17516821

[ref74] Niedenthal P. M. (2007). Embodying emotion. Science 316, 1002–1005. doi: 10.1126/science.1136930 17510358

[ref07] O’Keefe D. J. (2003). Colloquy: Should familywise alpha be adjusted? Against familywise alpha adjustment. Hum. Commun. Res. 29, 431–447.

[ref75] Olszanowski M. Wróbel M. Hess U. (2020). Mimicking and sharing emotions: a re-examination of the link between facial mimicry and emotional contagion. Cognit. Emot. 34, 367–376. doi: 10.1080/02699931.2019.1611543, PMID: 31072246

[ref76] Palinkas L. A. Browner D. (1995). Effects of prolonged isolation in extreme environments on stress, coping, and depression. J. Appl. Soc. Psychol. 25, 557–576. doi: 10.1111/j.1559-1816.1995.tb01599.x

[ref77] Passardi S. Peyk P. Rufer M. Wingenbach T. S. H. Pfaltz M. C. (2019). Facial mimicry, facial emotion recognition and alexithymia in post-traumatic stress disorder. Behav. Res. Ther. 122:103436. doi: 10.1016/j.brat.2019.103436 31557692

[ref78] Peter-Ruf C. Kirmse U. Pfeiffer S. Schmid M. Wilhelm F. In-Albon T. (2017). Emotion regulation in high and low socially anxious individuals: an experimental study investigating emotional mimicry, emotion recognition, and self-reported emotion regulation. J. Depress. Anxiety Disord. 1, 17–26. doi: 10.36959/362/469

[ref79] Prochazkova E. Kret M. E. (2017). Connecting minds and sharing emotions through mimicry: a neurocognitive model of emotional contagion. Neurosci. Biobehav. Rev. 80, 99–114. doi: 10.1016/j.neubiorev.2017.05.013, PMID: 28506927

[ref80] Rockwell D. A. Hodgson M. G. Beljan J. R. Winget C. M. (1976). Psychologic and psychophysiologic response to 105 days of social isolation. Aviat. Space Environ. Med. 47, 1087–1093. PMID: 985284

[ref81] Ruys K. I. Stapel D. A. (2008). Emotion elicitor or emotion messenger? Subliminal priming reveals two faces of facial expressions. Psychol. Sci. 19, 593–600. doi: 10.1111/j.1467-9280.2008.02128.x 18578850

[ref82] Sato W. Fujimura T. Kochiyama T. Suzuki N. (2013). Relationships among facial mimicry, emotional experience, and emotion recognition. PLoS One 8:e57889. doi: 10.1371/journal.pone.0057889, PMID: 23536774 PMC3607589

[ref83] Schiano Lomoriello A. Maffei A. Brigadoi S. Sessa P. (2021). Altering sensorimotor simulation impacts early stages of facial expression processing depending on individual differences in alexithymic traits. Brain Cogn. 148:105678. doi: 10.1016/j.bandc.2020.105678, PMID: 33454594

[ref84] Schneider K. G. Hempel R. J. Lynch T. R. (2013). That “poker face” just might lose you the game! The impact of expressive suppression and mimicry on sensitivity to facial expressions of emotion. Emotion 13, 852–866. doi: 10.1037/a0032847 23795586

[ref85] Segrin C. (2000). Social skills deficits associated with depression. Clin. Psychol. Rev. 20, 379–403. doi: 10.1016/S0272-7358(98)00104-4 10779900

[ref86] Seibt B. Mühlberger A. Likowski K. U. Weyers P. (2015). Facial mimicry in its social setting. Front. Psychol. 6:1122. doi: 10.3389/fpsyg.2015.01122, PMID: 26321970 PMC4531238

[ref87] Sessa P. Schiano Lomoriello A. Duma G. M. Mento G. De Stefani E. Ferrari P. F. (2022). Degenerate pathway for processing smile and other emotional expressions in congenital facial palsy: an hdEEG investigation. Philos. Trans. R. Soc. B 377:20210190. doi: 10.1098/rstb.2021.0190, PMID: 36126673 PMC9489284

[ref88] Sessa P. Schiano Lomoriello A. Luria R. (2018). Neural measures of the causal role of observers' facial mimicry on visual working memory for facial expressions. Soc. Cogn. Affect. Neurosci. 13, 1281–1291. doi: 10.1093/scan/nsy095, PMID: 30365020 PMC6277745

[ref90] Sheldon K. M. Corcoran M. Sheldon M. (2021). Duchenne smiles as honest signals of chronic positive mood. Perspect. Psychol. Sci. 16, 654–666. doi: 10.1177/1745691620959831, PMID: 33577410

[ref91] Sloan D. M. Bradley M. M. Dimoulas E. Lang P. J. (2002). Looking at facial expressions: Dysphoria and facial EMG. Biol. Psychol. 60, 79–90. doi: 10.1016/s0301-0511(02)00044-3, PMID: 12270585

[ref92] Söderkvist S. Ohlén K. Dimberg U. (2018). How the experience of emotion is modulated by facial feedback. J. Nonverbal Behav. 42, 129–151. doi: 10.1007/s10919-017-0264-1, PMID: 29497224 PMC5816132

[ref93] Stel M. Vonk R. (2010). Mimicry in social interaction: benefits for mimickers, mimickees, and their interaction. Br. J. Psychol. 101, 311–323. doi: 10.1348/000712609X465424, PMID: 19646328

[ref08] Strack F. Martin L. L. Stepper S. (1988). Inhibiting and Facilitating Conditions of the Human Smile: A Nonobtrusive Test of the Facial Feedback Hypothesis. J. Pers. Soc. Psychol. 54, 768–777. doi: 10.1037/0022-3514.54.5.768 3379579

[ref95] Tarantili V. V. Halazonetis D. J. Spyropoulos M. N. (2005). The spontaneous smile in dynamic motion. Am. J. Orthod. Dentofac. Orthop. 128, 8–15. doi: 10.1016/j.ajodo.2004.03.042, PMID: 16027620

[ref96] Tassinary L. G. Cacioppo J. T. (2000). The Skeletomotor System: Surface Electromyography. New York, NY, US: Cambridge University Press.

[ref99] Tourangeau R. Ellsworth P. C. (1979). The role of facial response in the experience of emotion. J. Pers. Soc. Psychol. 37, 1519–1531. doi: 10.1037/0022-3514.37.9.1519 501520

[ref100] Tuck N. L. Adams K. S. Pressman S. D. Consedine N. S. (2017). Greater ability to express positive emotion is associated with lower projected cardiovascular disease risk. J. Behav. Med. 40, 855–863. doi: 10.1007/s10865-017-9852-0, PMID: 28455831

[ref101] Tuck N. L. Grant R. C. I. Jackson A. Brooks A. E. S. Consedine N. S. (2016a). Beyond self-report: performance measures of emotional competencies predict symptoms of depression and anxiety, physical symptoms, self-rated health, and immunoregulatory molecules. Ann. Behav. Med. 50, 823–835. doi: 10.1007/s12160-016-9809-5, PMID: 27325315

[ref102] Tuck N. L. Grant R. C. I. Sollers J. J. Booth R. J. Consedine N. S. (2016b). Higher resting heart rate variability predicts skill in expressing some emotions. Psychophysiology 53, 1852–1857. doi: 10.1111/psyp.12755, PMID: 27565951

[ref103] Usala P. D. Hertzog C. (1989). Measurement of affective states in adults. Evaluation of an adjective rating scale instrument. Res. Aging 11, 403–426. doi: 10.1177/0164027589114001 2623354

[ref104] van Baaren R. B. Holland R. W. Kawakami K. van Knippenberg A. (2004). Mimicry and prosocial behavior. Psychol. Sci. 15, 71–74. doi: 10.1111/j.0963-7214.2004.01501012.x 14717835

[ref105] Varcin K. J. Bailey P. E. Henry J. D. (2010). Empathic deficits in schizophrenia: the potential role of rapid facial mimicry. J. Int. Neuropsychol. Soc. 16, 621–629. doi: 10.1017/S1355617710000329, PMID: 20374674

[ref107] Vaughan K. B. Lanzetta J. T. (1981). The effect of modification of expressive displays on vicarious emotional arousal. J. Exp. Soc. Psychol. 17, 16–30. doi: 10.1016/0022-1031(81)90003-2

[ref09] Wagenmakers E. J. Beek T. Dijkhoff L. Gronau Q. F. Acosta A. Adams R. B.. (2016). Registered Replication Report: Strack, Martin, & Stepper (1988). Perspect. Psychol. Sci. 11, 917–928. doi: 10.1177/1745691616674458 27784749

[ref108] Wexler B. E. Levenson L. Warrenburg S. Price L. H. (1993). Decreased perceptual sensitivity to emotion-evoking stimuli in depression. Psychiatr Res 51, 127–138. doi: 10.1016/0165-1781(94)90032-9, PMID: 8022947

[ref109] White M. (1995). Preattentive analysis of facial expressions of emotion. Cognit. Emot. 9, 439–460. doi: 10.1080/02699939508408975

[ref111] Winkielman P. Cacioppo J. T. (2001). Mind at ease puts a smile on the face: psychophysiological evidence that processing facilitation elicits positive affect. J. Pers. Soc. Psychol. 81, 989–1000. doi: 10.1037/0022-3514.81.6.989, PMID: 11761320

[ref112] Winkielman P. Coulson S. Niedenthal P. (2018). Dynamic grounding of emotion concepts. Philos Trans R Soc B Biol Sci 373:20170127. doi: 10.1098/rstb.2017.0127, PMID: 29914995 PMC6015836

[ref113] Wood A. Lupyan G. Sherrin S. Niedenthal P. (2016). Altering sensorimotor feedback disrupts visual discrimination of facial expressions. Psychon. Bull. Rev. 23, 1150–1156. doi: 10.3758/s13423-015-0974-5, PMID: 26542827

[ref114] Yoon S. Kim H. S. Kim J. I. Lee S. Lee S. H. (2016). Reading simple and complex facial expressions in patients with major depressive disorder and anxiety disorders. Psychiatry Clin. Neurosci. 70, 151–158. doi: 10.1111/pcn.12369, PMID: 26522432

[ref115] Yoshimura S. Sato W. Uono S. Toichi M. (2015). Impaired overt facial mimicry in response to dynamic facial expressions in high-functioning autism Spectrum disorders. J. Autism Dev. Disord. 45, 1318–1328. doi: 10.1007/s10803-014-2291-7, PMID: 25374131

[ref117] Ziebell L. Collin C. Mazalu M. Rainville S. Weippert M. Skolov M. (2021). Electromyographic evidence of reduced emotion mimicry in individuals with a history of non-suicidal self-injury. PLoS One 15:e0243860. doi: 10.1371/journal.pone.0243860, PMID: 33370320 PMC7769269

[ref118] Zwick J. C. Wolkenstein L. (2017). Facial emotion recognition, theory of mind and the role of facial mimicry in depression. J. Affect. Disord. 210, 90–99. doi: 10.1016/j.jad.2016.12.022 28024224

